# An Updated Meta-Analysis of Laparoscopic Versus Open Repair for Perforated Peptic Ulcer

**DOI:** 10.1038/srep13976

**Published:** 2015-09-09

**Authors:** Chunhua Zhou, Weizhi Wang, Jiwei Wang, Xiaoyu Zhang, Qun Zhang, Bowen Li, Zekuan Xu

**Affiliations:** 1 Department of General Surgery, The First Affiliated Hospital of Nanjing Medical University, Nanjing, China; 2 Hangzhou First People’s Hospital, Hangzhou, China; 3 The Second People’s Hospital of Huaian, Huaian, China; 4 Collaborative Innovation Center For Cancer Personalized Medicine, Nanjing Medical University, Nanjing, China

## Abstract

Laparoscopic repair (LR) for perforated peptic ulcer (PPU) has been introduced since 1990. Although many studies comparing LR with open repair (OR) have been published, controversy remains regarding the clinical utility of laparoscopic techniques for the treatment of PPU. Thus, it is necessary for us to broaden our knowledge on this subject with the newly published articles. Twenty-four nonrandomized controlled studies (NRS) and five randomized controlled trails (RCTs) were included in our meta-analyses, which comprised 5,268 patients (1,890 in the LR group and 3,378 in the OR group). In the analysis of high quality NRS and RCTs, compared with OR, high quality evidence suggested that LR was associated with a lower incidence of overall postoperative complications; moderate evidence showed that the two procedures had the similar reoperation rate; based on the low quality evidence, LR had reduced hospital mortality and similar operative time; Moreover, LR was observed having the advantages of earlier resumption of oral intake, shorter hospital stay and less analgesic use, which were supported by very low evidence. All the evidences suggest that LR is better than OR for PPU, but more high-quality RCTs are still needed for further validation.

The management of perforated peptic ulcer (PPU) has evolved greatly in the past three decades. With the discovery of *Helicobacter pylori* (HP) and advances in ulcer medications, omental patch repair for the PPU, followed by eradication of HP and the administration of proton pump inhibitors has become the standard treatment in most centers^1^,^2^,^3^. Open surgery for PPU has several shortcomings, such as a long incision, postoperative pain and slow recovery. Reports on the laparoscopic procedure for PPU were first published in 1990. Nathanson *et al.*^4^ reported the laparoscopic suture closure of a perforated duodenal ulcer, and Mouret *et al.*^5^ described a sutureless technique in which an omental patch with fibrin glue was used to seal the perforation in five patients under laparoscopy. Compared with the open procedure, laparoscopic surgery was associated with better magnified visualization during the procedure, minimal incision, less postoperative pain and faster resumption of activity.

With the development of these techniques, an increasing number of surgeons have opted to use the laparoscopic procedure, and many studies that evaluated the effectiveness of this approach for PPU have been published. However, whether laparoscopic repair (LR) is better than open repair (OR) for PPU remains debatable^6^,^7^,^8^,^9^,^10^,^11^. Several authors have suggested that laparoscopic surgery is not superior to laparotomy due to a lack of direct tactile sense, longer operative times and difficulty in peritoneal cavity flushing. To resolve these disputes, several meta-analyses on the advantages of LR vs. OR have been published^12^,^13^,^14^. The first meta-analysis^12^, which was published in 2004, revealed that laparoscopic repair (LR) for PPU conferred superior short-term benefits only in terms of postoperative pain and wound morbidity. In 2005, another meta-analysis^13^, which included 2 randomized controlled trials (RCTs) and 13 nonrandomized studies (NRS), indicated that LR had the advantages of less analgesic use, a shorter hospital stay, a lower risk of wound infection and a lower mortality rate but a longer operating time and a greater occurrence of suture-site leakage. The latest meta-analysis^14^, which was published in 2013, included 4 RCTs with 289 patients and suggested that the two approaches had similar morbidity, mortality, and reoperation rates.

The conclusions of these meta-analyses were contradictory. To date, 11 additional NRS and one more RCT have compared laparoscopic repair with open repair for PPU. Therefore, we performed an updated meta-analysis to determine the relative effectiveness of laparoscopic repair for PPU.

## Materials and Methods

### Literature search

We searched the electronic databases (Medline, Embase, and Web of Knowledge) to identify the English articles that compared laparoscopic repair with open repair for PPU. Articles published between January 1994 and September 2014 were selected. The key words “(perforated peptic ulcer OR duodenal ulcer OR gastric ulcer) AND (laparoscopy OR laparoscopic) AND (open OR conventional)” were used. Additionally, we also reviewed the bibliographic reference lists of the retrieved articles to find the suitable articles.

### Inclusion and exclusion criteria

All of the published studies that compared the outcomes of laparoscopic repair and open repair for PPU were included in the analysis. The exclusion criteria included animal or laboratory studies and clinical trials without major outcomes. To avoid duplication of the data sources, studies with a similar patient population and studies from the same center were included only once.

### Data extraction

The data were extracted and critically appraised by two independent authors. The characteristics of the study and the patients were documented and were presented in a table format. The major outcome measures for the descriptive and quantitative analyses included operative time, first oral intake day, analgesic use, postoperative complications (including suture-site leakage, intra-abdominal abscess or collection, ileus, difficulty with gastric emptying, gastrointestinal bleeding, wound infection, pneumonia, pleural effusion, urinary tract infection, and burst abdomen), length of hospital stay, mortality and reoperation rate.

### Quality assessment of the studies

The Newcastle-Ottawa quality assessment scale for cohort studies^15^(Table S1), which is recommended in the Cochrane Handbook version 5.1.0, was used to evaluate the quality of the nonrandomized studies by two independent reviewers. Eight elements in this scale are used to assess patient population and selection, study comparability, follow-up, and outcome of interest. High-quality elements are awarded by adding a star, and the stars are summed to compare the study quality. The studies with 7 or more stars were considered high-quality studies. The results are presented in Table 1. Any discrepancies were resolved by a consensus between the reviewers. RCTs were evaluated based on individual components using the Cochrane Collaboration Risk of Bias tool. A ‘risk of bias’ table is available as part of the ‘table of the characteristics of the included studies.’ Six question-based entries are involved in this table. For each entry, there are three responses (‘Yes’ for a low risk of bias; ‘No’ for a high risk of bias; or ‘Unclear’). A ‘risk of bias summary’ presents all of the responses in a cross-tabulation of the studies according to each entry in the table (Table 2). Any discrepancies were resolved by a consensus between the reviewers.

### Level of evidence

The level of evidence was assessed by GRADE (Grades of Recommendation, Assessment, Development and Evaluation) approach, and the evidence profile was created by GRADEprofiler 3.6 software.

According to the GRADE system, the level of evidence was defined as: (1). High quality: Our confidence in the estimate of effect is unlikely changed by the further research; (2). Moderate quality: Our confidence in the estimate of effect may be changed by the further research; (3) Low quality: Our confidence in the estimate of effect is likely changed by the further research; (4) Very low quality: We are uncertain about the estimate of effect.

### Statistical analysis

We used weighted mean differences (WMDs) with 95% confidence intervals (CIs) to analyze the continuous variables in the same scale (i.e., operative time and first oral intake day). When the mean or standard deviation (SD) was not reported in the study, it was estimated according to the formulas proposed by Hozo *et al.*^16^. The dichotomous data were calculated using the relative risks (RRs). Random effects models were used because of the high heterogeneity of the studies (*P* < 0.1). Otherwise, fixed-effects models were used^17^,^18^. To better investigate the heterogeneity among the outcome variables, we constructed a meta-regression model to examine the year of publication (before and after 2004), patients’ country (Eastern or Western), study type (prospective or retrospective cohort study), and operative procedure (omental patch repair, mixture or not mentioned). Subgroup-analyses were performed based on the meta-regression results and the study characteristics of interest to investigate the possible explanations of the heterogeneity and to assess the potential effect of these factors on the outcomes. A Galbraith plot was used to identify the articles that were the major contributors to heterogeneity^19^. Additionally, Funnel plots and Egger’s linear regression test were used to assess publication bias^20^. All of the statistical calculations were completed using the STATA program (version 10.1, StataCorp LP, College Station, TX, USA). A two-tailed value of *P* < 0.05 was considered statistically significant.

## Results

### Characteristics of the studies

**Figure 1:**
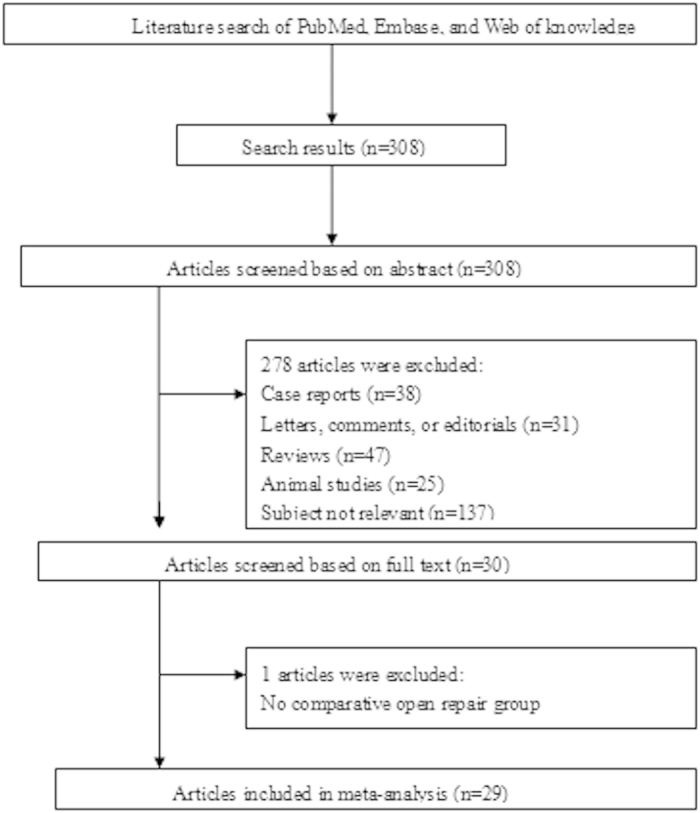
Articles identified with criteria for inclusion and exclusion.

The electronic search strategy identified 304 articles that mentioned laparoscopic and open repair for PPU. After screening the titles, abstracts, full texts, or a combination of these, we selected NRS^6^,^8^,^11^,^21^,^22^,^23^,^24^,^25^,^26^,^27^,^28^,^29^,^30^,^31^,^32^,^33^,^34^,^35^,^36^,^37^,^38^,^39^,^40^,^41^ and 5 RCTs^42^,^43^,^44^,^45^,^46^ (Table 3) based on the inclusion and exclusion criteria. Figure 1 shows a flow diagram that details the selection process.

All of the articles were published between 1995 and 2013. A total of 5,268 patients were analyzed in this study, of which 1,890 patients underwent LR and 3,378 patients received OR. Twenty-three studies were published by Western scholars, and only 6 studies were reported by Asian investigators. All the characteristics of the studies are shown in Table S2. In the NRS, the mean age of the patients ranged from 30–69 years, and the patients in the LR group were younger than those in the OR group. The distribution of gender was different in the two groups: the rate of LR was higher than that of OR in males. However, no significant differences were observed in the duration of acute symptoms, perforation size, shock on admission, and history of peptic ulcer, NSAID use and abdominal surgery. Therefore, these two procedures are comparable. No significant differences were found among the RCTs (Table S3).

After quality assessment, 15 NRS were identified as high quality studies (≥7 scores). To get the more reliable conclusions, we focused on the results of the high quality NRS and RCTs. The analyses of all NRS were still presented in the supplemental information (Table S4).

### Intraoperative and postoperative findings

**Figure 2:**
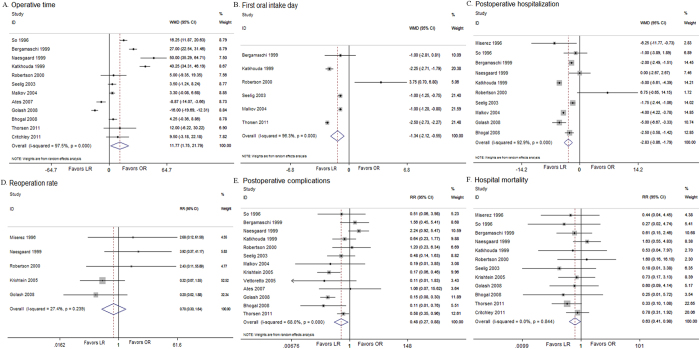
Analysis of high quality NRS comparing (A) operative time, (B) first oral intake, (C) postoperative hospital stay, (D) reoperation rate, (E) postoperative complications, (F) hospital mortality.

In the high quality NRS, longer operative times were found in the LR group compared with the OR group (WMD, 11.77; 95% CI, 1.75, 21.79; *P* = 0.021) (Fig. 2A). Inter-study variability was confirmed by a significant heterogeneity test result (I^2^ = 97.5%, *P* < 0.001). The outcomes indicated that the patients who underwent LR resumed a normal diet earlier than the patients who underwent OR (WMD, −1.34; 95% CI, −2.12, −0.55; *P* < 0.001) (Fig. 2B). However, significant heterogeneity (I^2^ = 96.3%, *P* < 0.001) was observed. Postoperative pain was evaluated by counting the days of analgesic use or the dosage. The patients who underwent the laparoscopic procedure used fewer analgesics (days: WMD, −3.60; 95% CI, −5.50, −1.70; *P* < 0.001; dosage: WMD, −106.59; 95% CI, −124.01, −89.17; *P* < 0.001) (Table 4). However, significant heterogeneity was detected in the studies according to the number of days of analgesic use (I^2^ = 98.2%, *P* < 0.001). The meta-analysis of the high quality NRS demonstrated that postoperative hospitalization favored LR (WMD, −2.83; 95% CI, −3.86, −1.80; *P* < 0.001) (Fig. 2C), but significant heterogeneity was observed among the studies (I^2^ = 92.9%, *P* < 0.001). Significant differences were not found in the analyses of the RCTs. Moreover, the reoperation rate was similar between the two groups in the high quality NRS (RR, 0.70; 95% CI, 0.30, 1.64; *P* = 0.412) (Fig. 2D) and the RCTs (RR, 2.11; 95% CI, 0.50, 8.97; *P* = 0.313). No significant heterogeneity was detected among the studies (NRS: I^2^ = 27.4%, *P* = 0.239; RCTs: I^2^ = 53.4%, *P* = 0.313).

### Morbidity and mortality

The meta-analysis of the high quality NRS demonstrated a lower overall rate of postoperative complications in the LR group (RR, 0.49; 95% CI, 0.27, 0.88; *P* = 0.018) (Fig. 2E). However, significant heterogeneity was found among the studies (I^2^ = 68.0%, *P* < 0.001). In addition, the meta-analysis of the RCTs confirmed this result (RR, 0.48; 95% CI, 0.36, 0.65; *P* < 0.001), and indicated fewer minor surgical complications after LR (RR, 0.45; 95% CI, 0.30, 0.67; *P* < 0.001) (Table 5). In the analyses of the high quality NRS, a lower rate of wound infections (RR, 0.28; 95% CI, 0.14, 0.56, *P* < 0.001) and ileus (RR, 0.33; 95% CI, 0.13, 0.83, *P* = 0.018) after LR was found according to the subcategory analysis (Table S5). In the analyses of the RCTs, fewer wound infections were observed in the LR group (RR, 0.52; 95% CI, 0.30, 0.93, *P* = 0.027). Significant differences in hospital mortality were found between the LR and OR groups in the high quality NRS (RR, 0.63; 95% CI, 0.41, 0.98; *P* = 0.039) (Fig. 2F), but not in the RCTs (RR, 0.39; 95% CI, 0.12, 1.32; *P* = 0.131).

### Meta-regression

According to the Cochrane handbook, meta-regression should not be considered for meta-analyses of less than ten studies. Therefore, we only examined the outcome variables (operative time, postoperative hospitalization, and postoperative complications) in more than ten studies in a meta-regression model. The analyses indicated that year of publication and the operation procedure contributed to the heterogeneity (Table 6).

### Subgroup analysis

Subgroup analyses were performed using the factor of heterogeneity (year of publication and operation procedure), and the study characteristics of interest (study type and patient country) (Table 7). The longer operative time of LR was only observed in the studies before 2004 and the studies of European patients. We did not observe significant differences in postoperative hospitalization in studies with Asian patients. No significant differences were detected in the first oral intake day between the two groups in the studies before 2002, the prospective cohort studies, and the studies on omental patch repair. A reduced reoperation rate was found between the groups in studies after 2004. the reduced postoperative complications after LR were associated with the studies after 2004, the retroprospective cohort studies, and the studies with Asian patients. Regarding hospital mortality, we did not observe any significant differences in the subgroup analyses.

### Sensitivity and Publication Bias

**Figure 3:**
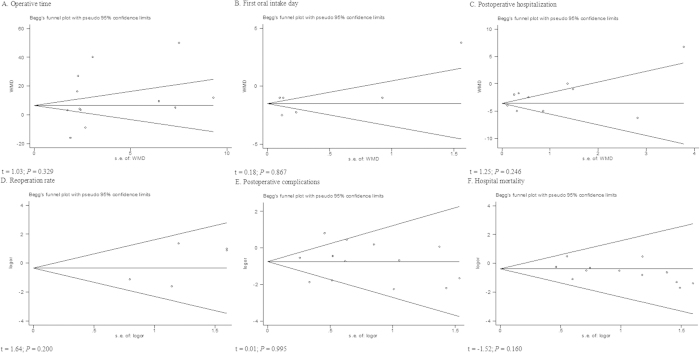
Funnel plots of each outcome. (**A**) operative time; (**B**) first oral intake; (**C**) postoperative hospital stay; (**D**) reoperation rate; (**E**) postoperative complications; (**F**) hospital mortality.

The funnel plots and Egger’s linear regression test were used to detect publication bias for each result. When the number of studies was small, there was a limitation in this test. So the funnel plots of analgesic medication were not showed. Six funnel plots were constructed for the outcomes of interest (Fig. 3). Symmetry was observed for most of the outcomes. None of the outcomes demonstrated significant publication bias. We used a Galbraith plot to determine which articles contributed to the heterogeneity (Fig. S1). Then, we excluded these articles and analyzed the pooled data from the remaining articles, and similar results were found.

### GRADE profile evidence

We assessed the quality of the primary outcomes of the RCTs and high quality NRS. The Table 8 shows the reasons for upgrade and downgrade and the GRADE quality of evidence for the primary outcomes.

## Discussion

Since the first introduction of LR for PPU in 1990, this approach has been widely accepted because it is minimally invasive. Many studies that compared laparoscopic repair with open repair for PPU have been published in the past few decades; however, a consensus on the best approach has not been reached. To resolve these debates, we performed this updated meta-analysis.

To summarize the highest quality of data from studies that compared LR and OR, we analyzed all of the available studies that met the inclusion criteria, which resulted in 24 NRS and 5 RCTs. Then, we used NOS and the Cochrane Risk of Bias Tool to evaluate the quality of the NRS and RCTs, respectively, and 15 NRS was regarded as high quality studies. After comparing the clinical characteristics between the two groups in the NRS, no significant differences were found in the duration of acute symptoms, perforation size, shock on admission, or a history of peptic ulcer, NSAID use and abdominal surgery. Nevertheless, the patients in the LR group were younger, and this group included more male patients. Because young people have better recovery after surgery, the results may demonstrate bias toward the laparoscopic approach. In the RCTs, the characteristics were similar between the two groups. According to the recommendations in the Cochrane Handbook, combining evidence from NRS and RCTs is not recommended. Therefore, we presented the analyses of the NRS and RCTs separately. Because high quality NRS (≥7 scores) and RCTs could provide us more reliable results, our final conclusions were dependent on both of them.

Because high heterogeneity was found in most of the outcomes, it was important to reduce the influence of the heterogeneity. We first performed a meta-regression and found that the year of publication was a factor that led to high heterogeneity. Then, subgroup analyses of the NRS were used to minimize the effects of the heterogeneity. However, high heterogeneity remained. Therefore, we considered that different levels of laparoscopic expertise, different laparoscopic equipment, and learning curve issues may have been potential sources of heterogeneity. Finally, we excluded the articles that were regarded as contributors to heterogeneity according to a Galbraith plot, and we analyzed the pooled data from the remaining articles to determine whether the same conclusions could be reached with low heterogeneity. We believe that our results are reliable and the conclusions are based on the best available data in the literature.

The meta-analysis of the NRS demonstrated that LR was associated with a little longer operative times than OR. Peritoneal cavity lavage under laparoscopy is difficult due to the limited operating space, and this factor may contribute to the prolonged duration. Because laparoscopy is a new technique, the lack of surgical experience with this novel approach may be another contributing factor. However, in the analysis of the RCTs, no significant differences in the operative times were found between the two approaches. Interestingly, in the subgroup-analysis, longer operative times were found in the LR group in the articles that were published before 2004, but similar operative times were observed in the studies that were published after 2004. This result indicates that the operative time of LR is gradually decreasing over time, which has similarly been proposed in a previous meta-analysis^13^. A feasible explanation is that the accumulation of laparoscopic expertise and technological advancements in equipment may shorten the operating time for LR. Moreover, several studies found that the operative time of LR was lower than that of OR^11^,^41^,^44^. A lower operative time is associated with less exposure to anesthesia and CO_2_ pneumoperitoneum, which may benefit patients in postoperative recovery. To date, the difference in operative time is minimal between LR and OR, which suggests that the two procedures may have similar operative times. The disadvantage of a longer operative time using the laparoscopic procedure may no longer apply.

The feeling of pain is subjective; therefore, we used the days of analgesic use or the dosages to estimate the level of pain in patients. Minimally invasive surgery results in a less painful recovery, and the analysis of the NRS confirmed this result. However, the meta-analysis of the RCTs did not reach the same conclusion but indicated less analgesic use in the LR group. This result may be explained by the relatively small sample size in the RCTs. However, the un-blinded nature of these studies regarding patient care may have led to an overestimation of the benefits of LR. In general, patents may suffer less pain after LR, but further investigations are needed.

The present analysis of the NRS demonstrated faster resumption of a normal diet and shorter hospital stays in the LR group. In addition, the outcomes of the RCTs were in favor of LR, but significant differences between LR and OR were not observed. LR is associated with minimal gastrointestinal interference and less pain; therefore, this approach has a lower impact on the body and patients may resume function of the gastrointestinal tract and daily activities earlier than patients who undergo OR. In the subgroup-analysis, significant differences were found in the studies after 2004 but not in the articles before 2004. This phenomenon may be due to the same reason as the decreased operative time.

Because of better visualization of the peritoneal cavity and minimal invasion, LR has the advantage of fewer postoperative complications, and this important factor may influence the surgeon’s choice of operative procedure. In the subcategory analysis, fewer wound infections were found in the LR groups in both the NRS and the RCTs because a large upper-abdominal incision is not necessary with the LR approach. The small incision by LR may reduce the occurrence of incisional hernia. This trend was found in the analysis of the RCTs but did not reach the statistical significance.

CO_2_ pneumoperitoneum is very important for laparoscopic procedures. However, the increased intra-abdominal pressure with CO_2_ pneumoperitoneum is associated with an increased risk of bacteremia and sepsis, and increased bacterial translocation from the peritoneal cavity into the bloodstream may cause pneumonia. In the previous studies, pneumonia has been found to occur more often in patients who undergo LR than OR even when the operative times were similar in both groups^26^. The present analysis demonstrated a similar pneumonia rate between the LR group and OR group. Lying in bed for a long period of time may increase the risk of pneumonia. Patients who undergo a laparoscopic operation experience less trauma, which suggests that these patients may get out of bed and resume normal activities earlier than patients who undergo OR. This benefit of LR may neutralize the disadvantages of CO_2_ pneumoperitoneum. Further validations are needed.

Because laparoscopic suture and peritoneal cavity lavage are technically more difficult, suture-site leakage and intra-abscess occur more frequently after LR. However, the present analysis did not demonstrate any significant differences between the two approaches. Laparoscopic surgery allows the surgeon to explore the peritoneal cavity with minimal trauma; therefore, fewer adhesions and a lower risk of postoperative ileus are several advantages of LR, but these benefits were not confirmed in this analysis. Similar incidences of urinary tract infection, difficulty with gastric emptying, gastrointestinal bleeding, pleural effusion and burst abdomen were found between the two groups; however, these symptoms were investigated in only a few studies.

Most of the variables in the subcategory analyses did not significantly vary between the LR and OR groups; however, a favorable trend toward LR was found. When we pooled the data together, reduced minor surgical complications after LR were found in RCTs and LR was also associated with a lower overall rate of complications in both the NRS and RCTs. In the sub-group analysis, fewer postoperative complications were observed in the LR group in studies after 2004 but not in those before 2004, which may be explained by increased laparoscopic expertise and improved instruments. The benefits of LR, such as a lower incidence of postoperative complications, were not found in the prospective cohort studies. After carefully reviewing these articles, we found that half of the prospective studies were published before 2004, which may explain this finding. A favorable trend toward LR was observed in the studies of Asian patients. Because only two high quality NRS on Asian patients were published, the relatively small sample size may have affected the results. Reduced postoperative morbidity after laparoscopic surgery was identified in both the NRS and RCTs, so we believed the result was reliable.

The main cause for reoperation following surgery is suture-site leakage. The laparoscopic approach may be associated with a higher reoperation rate because laparoscopic suturing is more complicated, especially when the edges of the perforation are infiltrated and friable. Using laparoscopic techniques, it is more difficult to properly tie knots; therefore, sutures may be easily torn. In a meta-analysis that was published in 2004^12^, a significantly higher reoperation rate was observed after LR. Nevertheless, in our analyses of NRS and RCTs, the reoperation rate was similar between the LR and OR approaches. Because a similar rate of suture-site leakage was found in our analysis, this result is reasonable. This outcome indicates that LR has become safer with improvements in the skill of surgeons and laparoscopic instruments.

The present analysis demonstrated a lower mortality rate in the LR group in the NRS. The results of the present study suggest that LR is a minimally invasive method that may decrease mortality. In patients with PPU, mortality is associated with sepsis and inflammation. Because inflammation has been alleviated after elective laparoscopic surgery^47^,^48^, this minimally invasive approach for PPU, which is an emergency condition, is correlated more closely with patient risk factors than surgical complications^49^. Thus, the selection bias of patients may lead to a higher mortality rate after OR. And in the analysis of RCTs, the mortality was similar between the two groups. Therefore, more RCTs are needed to confirm these findings.

In the sub-group analysis, the studies on omental patch repair did not demonstrate benefits of the laparoscopic procedure in most patients. Omental patch repair under laparoscopy is more difficult, which may affect operative outcomes. However, three of the four articles in the analysis were published before 2004, which may have influenced the results. Therefore, more data are needed to determine whether laparoscopic omental patch repair is a better choice for PPU patients.

We also used the GRADE system to assess the level of evidence. In high quality NRS, the quality of the outcomes was low (postoperative complications and hospital mortality) and very low (all the other results). In RCTs, the quality of the results was low (operative time, postoperative hospitalization and analgesic injection), moderate (first oral intake, reoperative rate and hospital mortality), and high (postoperative complications). High heterogeneity and few events might be the reasons.

In conclusion, combining the results of RCTs and NRS, LR is a feasible and safe option for PPU. Compared with OR, LR are associated with earlier resumption of oral intake, shorter hospital stay, less analgesic use, lower wound infections and the reduced incidence of overall postoperative complications and hospital mortality. Moreover, the reoperation rate and the operative time are similar between the two groups, However, further high-quality multicenter RCTs are needed to confirm the benefits of LR.

## Additional Information

**How to cite this article**: Zhou, C. *et al.* An Updated Meta-Analysis of Laparoscopic Versus Open Repair for Perforated Peptic Ulcer. *Sci. Rep.* **5**, 13976; doi: 10.1038/srep13976 (2015).

## Supplementary Material

Supplementary Information

## Figures and Tables

**Table 1:** Quality assessment of the nonrandomized studies base on the Newcastle-Ottawa quality assessment scale.

References	**selection**				**comparability**	**outcome**			score
	**1**	**2**	**3**	**4**	**5**	**6**	**7**	**8**	
Matsuda *et al.* (21)	*		*	*		*			4
Johansson *et al.* (22)	*	*	*	*		*			5
Miserez *et al.* (23)	*	*	*	*	**^1^,^4^	*			7
So *et al.* (24)	*	*	*	*	**^1^,^2^,^3^	*	*	*	9
Bergamaschi *et al.* (25)	*	*	*	*	*^1^	*	*	*	8
Naesgaard *et al.* (26)	*	*	*	*	**^1^,^2^,^3^	*			7
Katkhouda *et al.* (27)	*	*	*	*	**^1^,^3^	*	*	*	9
Kok *et al.* (28)	*	*	*	*	*^1^	*			6
Robertson *et al.* (29)	*	*	*	*	**^1^,^2^,^3^	*	*	*	9
Michelet *et al.* (30)	*	*	*	*		*			5
Lee *et al.* (31)	*	*	*	*		*			5
Mehendale *et al.* (32)	*	*	*	*	*^1^	*			6
Seelig *et al.* (33)	*	*	*	*	**^1^,^2^,^3^,^4^	*			7
Malkov *et al.* (34)	*		*	*	**^1^,^3^	*	*	*	8
Krishtein *et al.* (35)	*	*	*	*	**^1^,^2^,^3^	*			7
Vettoretto *et al.* (36)	*	*	*	*	**^1^,^2^,^3^	*			7
Lunevicius *et al.* (37)	*	*	*	*	*^1^	*			6
Ates *et al.* (11)	*	*	*	*	**^1^,^3^,^4^	*	*	*	9
Golash *et al.* (38)	*	*	*	*	**^1^,^2^,^3^	*			7
Bhogal *et al.* (8)	*	*	*	*	**^1^,^3^,^4^	*			7
Thorsen *et al.* (39)	*	*	*	*	**^1^,^2^,^3^	*			7
Kuwabara *et al.* (40)	*	*	*	*	*^1^	*			6
Critchley *et al.* (6)	*	*	*	*	**^1^,^2^	*			7
Dominguez-Vega *et al.* (41)	*	*	*	*	*^1^	*			6

^1^age and sex.

^2^ASA, American Society of Anesthesiology classification.

^3^duration of perforation.

^4^MPI, Mannheim Peritonitis-Index to score peritonitis.

**Table 2:** Quality assessment of the randomized controlled studies based on the Cochrane Risk of Bias Tool.

**Author**	**Year**	**Sequence generation**	**Allocation concealment**	**Blinding of participants, personnel and outcome**	**Incomplete outcome data**	**Selective outcome reporting**	**Other sources of bias**
Lau *et al.* (42)	1996	+	+	?	−	+	+
Lau *et al.* (43)	1998	+	?	?	?	+	+
Siu *et al.* (44)	2002	+	+	−	+	+	+
Bertleff *et al.* (45)	2009	+	+	−	+	+	+
Schietroma *et al.* (46)	2013	+	+	?	+	+	+

+: Low risk of bias?: Unclear −: High risk of bias.

**Table 3:** Details of the articles included in the meta-analysis.

References	Year	Country	Journal	**Sample size**		Type of the study
				**LR**	**OR**	
Matsuda *et al.* (21)	1995	Japan	Annal Surg	11	4	Retrospective Cohort study
Johansson *et al.* (22)	1996	Sweden	Surg Endosc	10	17	Retrospective Cohort study
Miserez *et al.* (23)	1996	Germany	Surg Endosc	18	16	Retrospective Cohort study
So *et al.* (24)	1996	Singapore	Surg Endosc	15	38	Retrospective Cohort study
Bergamaschi *et al.* (25)	1999	Norway	Surg Endosc	17	62	Prospective Cohort study
Naesgaard *et al.* (26)	1999	Norway	Eur J Surg	25	49	Retrospective Cohort study
Katkhouda *et al.* (27)	1999	America	Arch Surg	30	16	Prospective Cohort study
Kok *et al.* (28)	1999	Brunei	AM J Surg	11	20	Retrospective Cohort study
Robertson *et al.* (29)	2000	UK&Australia	Ann R Coll Surg	20	16	Prospective Cohort study
Michelet *et al.* (30)	2000	Italy	Eur J Surg	16	14	Retrospective Cohort study
Lee *et al.* (31)	2001	China	Br J Surg	155	219	Prospective Cohort study
Mehendale *et al.* (32)	2002	India	Indian J Gastroenterol	34	33	Prospective Cohort study
Seelig *et al.* (33)	2003	Germany	J Clin gastroenterology	24	31	Retrospective Cohort study
Malkov *et al.* (34)	2004	Azerbaijan	J AM COLL	42	40	Retrospective Cohort study
Krishtein *et al.* (35)	2005	Israel	Surg Endosc	68	66	Retrospective Cohort study
Vettoretto *et al.* (36)	2005	Italy	CHIRURGIA ITALIANA	10	10	Retrospective Cohort study
Lunevicius *et al.* (37)	2005	Lithuania	Surg Endosc	60	162	Retrospective Cohort study
Ates *et al.* (11)	2007	Turkey	J LAPAROENDOSC ADV A	17	18	Prospective Cohort study
Golash *et al.* (38)	2008	Oman	Omen medical J	95	57	Retrospective Cohort study
Bhogal *et al.* (8)	2008	UK	World J Surg	19	14	Prospective Cohort study
Thorsen *et al.* (39)	2011	Norway	J Gastrointest Surg	36	66	Retrospective Cohort study
Kuwabara *et al.* (40)	2011	Japan	World J Surg	836	2073	Retrospective Cohort study
Critchley *et al.* (6)	2011	UK	Ann R Coll Surg	53	89	Prospective Cohort study
Dominguez-Vega *et al.* (41)	2013	Spain	CIR ESP	60	52	Retrospective Cohort study
Lau *et al.* (42)	1996	China	Annal Surg	24	21	Randomized controlled trail
Lau *et al.* (43)	1998	China	AM J Surg	12	10	Randomized controlled trail
Siu *et al.* (44)	2002	China	Annal Surg	63	58	Randomized controlled trail
Bertleff *et al.* (45)	2009	Netherlands	World J Surg	52	49	Randomized controlled trail
Scheietroma *et al.* (46)	2013	Italy	J INVEST SURG	57	58	Randomized controlled trail

LR, laparoscopic repair; OR, open repair

**Table 4:** Overall results comparing LR with OR.

Items	Type	n^a^	WMD or RR 95% CI	**Test for Overall Effect**		**Test for Heterogeneity**	
				***Z***	***P***	***I*****^2^**	***P***
Operative time	NRS^b^	12	11.77 (1.75, 21.79)	2.30	**0.021**	97.5%	**<0.001**
	RCT	3	7.61 (−11.93, 27.15)	0.76	0.445	90.5%	**<0.001**
First oral intake day	NRS^b^	6	−1.34 (−2.12, −0.55)	3.34	**0.001**	96.3%	**<0.001**
	RCT	2	−0.27(−1.51, 0.97)	0.42	0.672	<0.1%	0.483
Postoperative hospitalization	NRS^b^	10	−2.83 (−3.86, −1.80)	5.37	**<0.001**	92.9%	**<0.001**
	RCT	3	−2.36 (−6.47, 1.74)	1.13	0.259	91.1%	**<0.001**
Reoperation rate	NRS^b^	5	0.70 (0.30, 1.64)	0.82	0.412	27.4%	0.239
	RCT	2	2.11 (0.50, 8.97)	1.01	0.313	53.4%	0.143
Postoperative complications	NRS^b^	13	0.49 (0.27, 0.88)	2.37	**0.018**	68.0%	**<0.001**
	RCT	4	0.48 (0.36, 0.65)	4.82	**<0.001**	<0.1%	0.596
Hospital mortality	NRS^b^	15	0.63 (0.41, 0.98)	2.07	**0.039**	<0.1%	0.844
	RCT	3	0.39 (0.17, 1.32)	1.51	0.131	<0.1%	0.951
Analgesic injection (days)	NRS^b^	3	−3.60 (−5.50, −1.70)	3.70	**<0.001**	98.2%	**<0.001**
	RCT	3	−2.74 (−5.62, 0.15)	1.86	0.063	93.3%	**<0.001**
Analgesic injection (mg)	NRS^b^	3	−106.59 (−124.01, −89.17)	11.99	**<0.001**	23.4%	0.271

CI, confidence interval; LR, laparoscopic repair; OR, open repair; RR, relative risks; WMD, weighed mean difference; NRS, non-randomized studies; RCT, randomzed controlled trails; data in bold, significant *P*-value.

^a^Number of comparisons.

^b^High quality NRS (≥7 scores).

**Table 5:** Analysis of postoperative complications comparing LR with OR by categories.

Surgical complications*	Type	n^a^	RR 95% CI	**Test for Overall Effect**		**Test for Heterogeneity**	
				***Z***	***P***	***I*****^2^**	***P***
Major	NRS^b^	7	0.62 (0.31, 1.24)	1.34	0.181	<0.1%	0.689
	RCT	4	0.69 (0.28, 1.71)	0.80	0.422	22.0%	0.279
Minor	NRS^b^	13	0.43 (0.18, 1.06)	1.84	0.066	68.3%	**<0.001**
	RCT	4	0.45 (0.30, 0.67)	3.94	**<0.001**	<0.1%	0.672

CI, confidence interval; LR, laparoscopic repair; OR, open repair; RR, relative risks; NRS, non-randomized studies; RCT, randomzed controlled trails; data in bold, significant *P*-value.

^*^Definitions: mjor surgical complications: anastomotic leak or fistula, complications that required reoperation or resulted in hospital death and all intra-abdominal collections; minor surgical complications: wound complications, any bleeding event, pancreatitis, pneumonia, urinary tract infection, burst abdomen, ileus, gastric emptying difficulty, and anastomotic stricture.

^a^Number of comparisons.

^b^High quality NRS (≥7 scores).

**Table 6:** Meta-regression analysis of the high quality NRS.

**Variable**	**Coefficient**	**Standard error**	***P*** **value**	**95% CI**
Operative time				
Year of publication	32.400	12.368	**0.034**	3.154 to 61.646
Study type	15.811	13.661	0.285	−16.493 to 48.115
procedure	15.549	10.363	0.177	−8.955 to 40.052
Country of patients	16.004	22.112	0.493	−36.282 to 68.290
Postoperative hospital stay				
Year of publication	3.649	2.745	0.241	−3.406 to 10.705
Study type	1.583	2.052	0.475	−3.692 to 6.857
procedure	2.534	1.699	0.196	−1.832 to 6.901
Country of patients	4.406	4.000	0.321	−5.878 to 14.689
Postoperaticve complications				
Year of publication	1.367	0.382	**0.007**	0.487 to 2.248
Study type	0.523	0.552	0.371	−0.750 to 1.796
procedure	0.842	0.342	**0.039**	0.054 to 1.630
Country of patients	0.510	0.382	0.500	−1.157 to 2.177

NRS, non-randomized studies; Data in bold, significant *P*-value.

**Table 7:** Subgroup-analyses of the high quality NRS by study quality, publish year, study type and type of the procedures.

Items	n^a^	RR or WMD 95% CI	**Test for Overall Effect**		**Test for Heterogeneity**	
			***Z***	***P***	***I*****^2^**	***P***
**Operative time**						
Before 2004	6	23.34 (10.97, 35.72)	3.70	**<0.001**	96.0%	**<0.001**
After 2004	6	−0.62 (−9.54, 8.30)	0.14	0.892	93.9%	**<0.001**
Prospective cohort study	6	13.03 (−3.00, 29.06)	1.59	0.111	97.5%	**<0.001**
Retrospective cohort study	6	10.17 (−2.37, 22.72)	1.59	0.112	97.1%	**<0.001**
Omental patch repair	4	11.42 (−15.12, 37.96)	0.84	0.399	98.9%	**<0.001**
European patients	10	14.23 (3.88, 24.58)	2.70	**0.007**	96.6%	**<0.001**
Asian patients	2	0.10 (−31.50, 31.71)	0.01	0.995	99.2%	**<0.001**
**Postoperative hospitalization**						
Before 2004	7	−2.09 (−3.73, −0.44)	1.56	**0.013**	92.8%	**<0.001**
After 2004	3	−3.75 (−4.89, −2.61)	6.44	**<0.001**	76.9%	**0.013**
Prospective cohort study	4	−2.52 (−4.65, −0.41)	2.33	**0.020**	95.4%	**<0.001**
Retrospective cohort study	6	−2.84 (−4.35, −1.33)	3.68	**<0.001**	90.3%	**<0.001**
Omental patch repair	4	−3.08 (−5.47, −0.68)	2.52	**0.012**	81.8%	**0.001**
European patients	8	−2.69 (−3.84, −1.55)	4.62	**<0.001**	94.2%	**<0.001**
Asian patients	2	−3.18 (−7.08, 0.72)	1.60	0.110	81.9%	**0.019**
**First oral intake**						
Before 2004	4	−0.94 (−2.10, 0.22)	1.59	0.112	90.8%	**<0.001**
After 2004	2	−1.75 (−3.22, −0.28)	2.33	**0.020**	98.9%	**<0.001**
Prospective cohort study	3	−0.24 (−2.93, 2.45)	0.18	0.860	87.4%	**<0.001**
Retrospective cohort study	3	−1.50 (−2.47, −0.53)	3.03	**0.002**	98.2%	**<0.001**
Omental patch repair	2	0.55 (−5.32, 6.42)	0.18	0.854	93.1%	**<0.001**
European patients	6	−1.34 (−2.12, −0.55)	3.34	**0.001**	96.3%	**<0.001**
Asian patients	1	/	/	/	/	/
**Reoperative rate**						
Before 2004	3	3.08 (0.62, 15.36)	1.37	0.170	<0.1%	0.966
After 2004	2	0.28 (0.08, 0.99)	1.98	**0.048**	<0.1%	0.730
Prospective cohort study	1	/	/	/	/	/
Retrospective cohort study	4	0.61 (0.25, 1.51)	1.06	0.289	38.1%	0.183
Omental patch repair	2	0.49 (0.10, 2.27)	0.92	0.359	38.1%	0.204
European patients	4	0.94 (0.36, 2.47)	0.12	0.901	24.7%	0.263
Asian patients	1	/	/	/	/	/
**Postoperative complications**						
Before 2004	6	1.03 (0.64, 1.65)	0.10	0.918	18.7%	0.292
After 2004	7	0.24 (0.12, 0.51)	3.73	**<0.001**	59.6%	**0.022**
Prospective cohort study	5	0.65 (0.35, 1.18)	1.43	0.154	31.5%	0.211
Retrospective cohort study	8	0.39 (0.18, 0.86)	2.33	**0.020**	75.9%	**<0.001**
Omental patch repair	4	0.43 (0.15, 1.21)	1.60	0.110	66.6%	**0.029**
European patients	11	0.58 (0.32, 1.05)	1.79	0.073	57.4%	**0.009**
Asian patients	2	0.18 (0.10, 0.33)	5.55	**<0.001**	14.1%	0.281
**Hospital mortality**						
Before 2004	7	0.74 (0.39, 1.43)	0.89	0.375	<0.1%	0.657
After 2004	5	0.56 (0.31, 1.00)	1.96	0.050	<0.1%	0.803
Prospective cohort study	5	0.71 (0.37, 1.40)	0.99	0.325	<0.1%	0.905
Retrospective cohort study	7	0.58 (0.32, 1.03)	1.86	0.062	<0.1%	0.495
Omental patch repair	4	0.62 (0.20, 1.96)	0.81	0.416	<0.1%	0.806
European patients	10	0.65 (0.42, 1.03)	1.83	0.067	<0.1%	0.743
Asian patients	2	0.43 (0.09, 2.15)	1.02	0.306	<0.1%	0.644

CI, confidence interval; RR, relative risks; WMD, weighed mean difference; data in bold, significant *P*-value.

^a^Number of comparisons.

**Table 8:** GRADE profile evidence of the included studies.

Items	n^a^	Type	Risk of bias	Quality assessment				No. of patients		Effect		Quality	Importance
				Inconsistency	Indirectness	Imprecision	Other considerations	LR	OR	Relative 95% CI	Absolute		
Operative time	12	NRS^b^	No Serious	Serious^1^	No serious indirectness	No serious imprecision	None	393	496	—	WMD 11.77 higher (1.75 to 21.79 higher)	≈OOO Very low	Important
	3	RCT	No Serious	Serious^1^	No serious indirectness	Serious^2^	None	144	137	—	WMD 7.61 higher (11.93 lower to 27.15 higher)	≈≈OO Low	Important
First oral intake	6	NRS^b^	No Serious	Serious^1^	No serious indirectness	No serious imprecision	None	169	231	—	WMD 1.34 lower (2.12 to 0.55 lower)	≈OOO Very low	Important
	2	RCT	No Serious	No serious inconsistency	No serious indirectness	Serious^2^	None	87	79	—	WMD 0.27 lower (1.51 lower to 0.97 higher)	≈≈≈O Moderate	Important
Postoperative hospitalization	10	NRS^b^	No Serious	Serious^1^	No serious indirectness	No serious imprecision	None	305	339	—	WMD 2.38 lower (3.86 to 1.80 lower)	≈OOO Very low	Important
	3	RCT	No Serious	Serious^1^	No serious indirectness	Serious^2^	None	144	137	—	WMD 2.36 lower (6.47 lower to 1.74 higher)	≈≈OO Low	Important
Reoperative rate	5	NRS^b^	No Serious	No serious inconsistency	No serious indirectness	Serious^3^	None	7/226 (3.1%)	10/214 (4.7%)	RR 0.70 (0.30, 1.64)	14 fewer per 1000 (from 33 fewer to 30 more)	≈OOO Very low	Important
	2	RCT	No Serious	No serious inconsistency	No serious indirectness	Serious^2^	None	5/87 (5.7%)	2/79 (2.5%)	RR 2.11 (0.50, 8.97)	28 more per 1000 (from 13 fewer to 202 more)	≈≈≈O Moderate	Important
Postoperative complications	13	NRS^b^	No Serious	Serious^1^	No serious indirectness	No serious imprecision	Strong association^4^	51/418 (12.2%)	144/319 (42.5%)	RR 0.49 (0.27 to 0.88)	217 fewer per 1000 (from 51 fewer to 310 fewer)	≈≈OO Low	Critical
	4	RCT	No Serious	No serious inconsistency	No serious indirectness	No serious imprecision	Strong association^4^	44/196 (22.4%)	88/186 (47.3%)	RR 0.48 (0.36 to 0.65)	246 fewer per 1000 (from 166 fewer to 303 fewer)	≈≈≈≈ High	Critical
Hospital mortality	15	NRS^b^	No Serious	No serious inconsistency	No serious indirectness	No serious imprecision	None	25/489 (5.1%)	63/588 (10.7%)	RR 0.63 (0.41 to 0.98)	40 fewer per 1000 (from 2 fewer to 63 fewer)	≈≈OO Low	Critical
	3	RCT	No Serious	No serious inconsistency	No serious indirectness	Serious^2^	None	3/139 (2.2%)	8/128 (6.3%)	RR 0.39 (0.17 to 1.32)	38 fewer per 1000 (from 52 fewer to 20 more)	≈≈≈O Moderate	Critical
Analgesic injection (days)	3	NRS^b^	No Serious	Serious^1^	No serious indirectness	Serious^2^	None	149	104	—	WMD 3.60 lower (5.50 to 1.70 lower)	≈OOO Very low	Important
	3	RCT	No Serious	Serious^1^	No serious indirectness	Serious^2^	None	139	128	—	WMD 2.74 lower (5.62 lower to 0.15 higher)	≈≈OO Low	Important
Analgesic injection (mg)	3	NRS^b^	No Serious	No serious inconsistency	No serious indirectness	Serious^2^	None	52	116	—	WMD 106.59 lower(124.01 to 89.17 lower)	≈OOO Very low	Important

NRS, non-randomized studies; RCT, randomzed controlled trails; LR, laparoscopic repair; OR, open repair; RR, relative risks; CI, confidence interval.

^a^Number of comparisons.

^b^High quality NRS (≥7 scores).

^1^High heterogeneity.

^2^Few events.

^3^Events present wild confidence intervals.

^4^Large magnitude of effect (RR <0.5 with no plausible confounders).
