# Supplementary material for: An Updated Meta-Analysis of Laparoscopic Versus Open Repair for Perforated Peptic Ulcer
Source: Sci Rep. 2015 Sep 9;5:13976. doi: 10.1038/srep13976 (PMC4563564; doi:10.1038/srep13976)

**An Updated Meta-Analysis of Laparoscopic Versus Open Repair for Perforated Peptic Ulcer**

Chunhua Zhou^1, 2,†^, Weizhi Wang^1,†^, Jiwei Wang^1,†^, Xiaoyu Zhang^1^, ^3,†^, Qun Zhang^1^, Bowen Li^1^, Zekuan Xu^1, 4^*

Author’s Affiliations:

^1^ Department of General Surgery, The First Affiliated Hospital of Nanjing Medical University, Nanjing, China; ^2^Hangzhou First People’s Hospital, Hangzhou, China; ^3^The Second people’s Hospital of Huaian, Huaian, China; ^4^Collaborative Innovation Center For Cancer Personalized Medicine, Nanjing Medical University, Nanjing, China.

^†^These authors contribute equally to this work.

***Correspondence to:**

Zekuan Xu M.D. Ph.D., Department of General Surgery, First Affiliated Hospital of Nanjing Medical University, 300 Guangzhou Road, Nanjing, Jiangsu Province, 210029, China, Email: [xuzekuan@njmu.edu.cn](mailto:xuzekuan@njmu.edu.cn)

**Table S1.** Newcastle–Ottawa quality assessment scale﹡

| **Selection** |
| --- |
| (1) Representativeness of the exposed cohort |
| (a) Truly representative of the average ‘PPU patient’ in the community (1 star) |
| (b) Somewhat representative of the average ‘PPU patient’ in the community (1 star) |
| (c) Selected group of users (e.g. nurses, volunteers) |
| (d) No description of the derivation of the cohort |
| (2) Selection of the non-exposed cohort |
| (a) Drawn from the same community as the exposed cohort (1 star) |
| (b) Drawn from a different source |
| (c) No description of the derivation of the non-exposed cohort |
| (3) Ascertainment of exposure |
| (a) Secure record (e.g. surgical records) (1 star) |
| (b) Structured interview (1 star) |
| (c) Written self-report |
| (d) No description |
| (4) Demonstration that outcome of interest was not present at start of study |
| (a) Yes (1 star) |
| (b) No |
| **Comparability** |
| (1) Comparability of cohorts on the basis of the design or analysis |
| (a) Study controls for ‘ age, sex’ (1 star) |
| (b) Study controls for any additional factor (1 star) (ASA, size, degree of peritonitis etc.) |
| **Outcome** |
| (1) Assessment of outcome |
| (a) Independent blind assessment (1 star) |
| (b) Record linkage (1 star) |
| (c) Self-report |
| (d) No description |
| (2) Was follow-up long enough for outcomes to occur? |
| (a) Yes (‘2 months’) (1 star) |
| (b) No |
| (3) Adequacy of follow-up of cohorts |
| (a) Complete follow-up – all subjects accounted for (1 star) |
| (b) Subjects lost to follow-up unlikely to introduce bias – small number lost ‘5%’ or description provided of those lost (1 star) |
| (c) Follow-up rate ‘<95%’ and no description of those lost |
| (d) No statement |

﹡A study can be awarded a maximum of one star for each numbered item within the Selection and Outcome categories. A maximum of two stars can be given for Comparability. Underlined and quoted phrases are provided in the scale to allow for adjustment to particular studies. Italicized phrases indicate our interpretation of the question relevant to this study.

PPU,perforated peptic ulcer; ASA, American Society of Anesthesiology classification.

**Table S2.** Characteristics of the articles included in the meta-analysis

| **References** | **Type** | **Approach** | **Age (years)** | **Male (No.)** | **Duration of acute symptoms（h）** | **Shock on admission** | **History of peptic ulcer** | **History of NSAID** | **Previous abdominal surgeries** | **Perforation size(mm)** | **Boey score** | | | | **ASA** | | | |
| --- | --- | --- | --- | --- | --- | --- | --- | --- | --- | --- | --- | --- | --- | --- | --- | --- | --- | --- |
|  |  |  |  |  |  |  |  |  |  |  | 0 | 1 | 2 | 3 | Ⅰ | Ⅱ | Ⅲ | Ⅳ |
| Matsuda et al | NRS | LR | 39.8 (19-81) | 10 | 8.4 (4-21) | 0 |  |  |  |  |  |  |  |  |  |  |  |  |
|  |  | OR | 49.5 (33-66) | 4 | 14 (6-19) | 0 |  |  |  |  |  |  |  |  |  |  |  |  |
| Johansson et al | NRS | LR | 66 (45-87) |  |  |  |  |  |  |  |  |  |  |  | 2 | 5 | 2 | 10 |
|  |  | OR | 64 (26-85) |  |  |  |  |  |  |  |  |  |  |  | 7 | 4 | 4 | 11 |
| Miserez et al | NRS | LR | 50 (21-75) | 10 |  |  | 10 |  |  |  |  |  |  |  |  |  |  |  |
|  |  | OR | 42 (21-84) | 10 |  |  | 7 |  |  |  |  |  |  |  |  |  |  |  |
| So et al | NRS | LR | 46 (20-67) | 13 | >24h,1 | 0 |  |  |  |  |  |  |  |  | 12 | 3 | 0 |  |
|  |  | OR | 58 (16-84) | 27 | >24h,7 | 2 |  |  |  |  |  |  |  |  | 21 | 6 | 11 |  |
| Bergamaschi et al | NRS | LR | 69 (27-83) | 14 | 6 (3-24) | 1 |  | 4 | 1 |  | 9 | 7 | 1 | 0 | 2 | 6 | 5 | 4 |
|  |  | OR | 74 (30-91) | 26 | 5 (1-79) | 4 |  | 14 | 4 |  | 28 | 31 | 2 | 1 | 4 | 12 | 25 | 21 |
| Naesgaard et al | NRS | LR | 69 (37-84) | 10 | 10 (2-96) |  | 8 | 12 |  |  |  |  |  |  | 2 | 8 | 13 | 2 |
|  |  | OR | 70 (16-86) | 26 | 10 (1-96) |  | 12 | 8 |  |  |  |  |  |  | 5 | 17 | 21 | 5 |
| Katkhouda et al | NRS | LR | 42 (31-45) |  | 22 (12-33) | 6 | 16 |  |  |  |  |  |  |  |  |  |  |  |
|  |  | OR | 39 (29-47) |  | 27 (12-31) | 4 | 0 |  |  |  |  |  |  |  |  |  |  |  |
| Kok et al | NRS | LR | 39 | 10 |  |  |  |  |  |  |  |  |  |  |  |  |  |  |
|  |  | OR | 41 | 19 |  |  |  |  |  |  |  |  |  |  |  |  |  |  |
| Robertson et al | NRS | LR | 62 (17-88) | 11 | 16 (5-112) |  |  |  |  |  |  |  |  |  | 4 | 5 | 6 | 4 |
|  |  | OR | 55 (18-91) | 6 | 10 (4-72) |  |  |  |  |  |  |  |  |  | 6 | 4 | 4 | 2 |
| Mehendale et al | NRS | LR | 38 | 33 |  |  |  |  |  |  |  |  |  |  |  |  |  |  |
|  |  | OR | 34 | 31 |  |  |  |  |  |  |  |  |  |  |  |  |  |  |
| Seelig et al | NRS | LR | 49 (36-59) | 16 | 14 |  |  |  | 1 |  |  |  |  |  | 11 | 10 | 3 | 0 |
|  |  | OR | 60 (47-78) | 13 | 15.2 |  |  |  | 2 |  |  |  |  |  | 5 | 4 | 11 | 11 |
| Malkov et al | NRS | LR | 18-43 | 39 |  |  | 15 |  |  |  |  |  |  |  |  |  |  |  |
|  |  | OR | 18-44 | 40 |  |  | 14 |  |  |  |  |  |  |  |  |  |  |  |
| Krishtein et al | NRS | LR | 47.9 (17-90) | 24 | 14.7 |  |  | 6 | 10 |  |  |  |  |  | 30 | 25 | 7 | 3 |
|  |  | OR | 48.8 (19-82) | 29 | 17.4 |  |  | 7 | 9 |  |  |  |  |  | 26 | 32 | 7 | 4 |
| Vettoretto et al | NRS | LR | 40.6 (23-62) |  | 8.1 (4-15) | 0 | 3 | 0 | 4 | 4.9 |  |  |  |  |  |  |  |  |
|  |  | OR | 47.7 (32-59) |  | 9.3 (5-20) | 1 | 5 | 7 | 2 | 5.0 |  |  |  |  |  |  |  |  |
| Lunevicius et al | NRS | LR | 34.0 ± 14.2 | 55 |  |  | 12 |  | 5 | 4.1 ± 2.5 | 54 | 5 | 1 | 0 | 21 | 36 | 3 | 0 |
|  |  | OR | 43.8 ± 21.4 | 114 |  |  | 45 |  | 33 | 5.6 ± 2.3 | 114 | 18 | 22 | 8 | 45 | 73 | 25 | 14 |
| Ates et al | NRS | LR | 30.53 (19-60) | 17 | 6.41 |  | 3 | 2 |  | 5.82 |  |  |  |  |  |  |  |  |
|  |  | OR | 31.88 (17-57) | 15 | 6.94 |  | 2 | 3 |  | 6 |  |  |  |  |  |  |  |  |
| Bhogal et al | NRS | LR | 54.8 (32-82) | 13 | 19.1 (11-29) |  |  |  |  | 5.5 (2-9) |  |  |  |  |  |  |  |  |
|  |  | OR | 52.1 (34-78) | 11 | 19.8 (13-27) |  |  |  |  | 5 (3-7) |  |  |  |  |  |  |  |  |
| Thorsen et al | NRS | LR | 62 (29-95) | 11 | 5.8 (1.8-113) |  |  |  |  |  | 10 | 21 | 4 | 1 | 0 | 1 | 26 | 8 |
|  |  | OR | 71 (20-100) | 29 | 6.6 (1.4-116) |  |  |  |  |  | 12 | 33 | 17 | 4 | 0 | 1 | 38 | 25 |
| Kuwabara et al | NRS | LR | 51.4 ± 17.3 | 679 |  |  |  |  |  |  |  |  |  |  |  |  |  |  |
|  |  | OR | 57.9 ± 18.4 | 1553 |  |  |  |  |  |  |  |  |  |  |  |  |  |  |
| Critchley et al | NRS | LR | 54 (17-96) | 38 |  |  |  |  |  |  |  |  |  |  | 10 | 22 | 12 | 8 |
|  |  | OR | 60 (17-95) | 49 |  |  |  |  |  |  |  |  |  |  | 16 | 25 | 22 | 22 |
| Dominguez-Vega et al | NRS | LR | 38.5 (16-78) | 48 | 6 (1-72) |  |  |  |  | 5 (3-30) | 27 | 21 | 9 | 3 | 15 | 31 | 13 | 1 |
|  |  | OR | 57.5 (25-91） | 40 | 12 (1-168) |  |  |  |  | 5 (2-30) | 10 | 26 | 14 | 2 | 18 | 16 | 13 | 5 |
| Lau et al | RCT | LR | 52.3 ± 13.8 | 20 |  | 2 |  |  |  | 6 (1-20) |  |  |  |  |  |  |  |  |
|  |  | OR | 51.1 ± 19.7 | 17 |  | 3 |  |  |  | 5 (2-25) |  |  |  |  |  |  |  |  |
| Lau et al | RCT | LR |  |  | 13.5 |  |  |  |  |  |  |  |  |  |  |  |  |  |
|  |  | OR |  |  | 10 |  |  |  |  |  |  |  |  |  |  |  |  |  |
| Siu et al | RCT | LR | 53.8 ± 18.4 | 53 | >24h,1 | 2 | 11 | 14 |  | 5.2 ± 4.9 |  |  |  |  | 33 | 18 | 8 | 4 |
|  |  | OR | 56.1 ± 19.0 | 45 | >24h,6 | 3 | 15 | 12 |  | 4.7 ± 3.0 |  |  |  |  | 29 | 19 | 8 | 2 |
| Bertleff et al | RCT | LR | 66 ± 25.8 | 29 | 11 (17) |  |  |  |  | 10.0 (7.0) |  |  |  |  |  |  |  |  |
|  |  | OR | 59 ± 29.5 | 32 | 11 (19) |  |  |  |  | 7.0 (6.0) |  |  |  |  |  |  |  |  |
| Scheietroma et al | RCT | LR | 58.9 ± 12.5 | 35 |  |  |  |  |  |  |  |  |  |  | 18 | 28 | 11 | 0 |
|  |  | OR | 58.1 ± 12.5 | 36 |  |  |  |  |  |  |  |  |  |  | 19 | 27 | 12 | 0 |

LR, laparoscopic repair; OR, open repair; NRS, non-randomized studies; RCT, randomzed controlled trails; ASA, American Society of Anesthesiology classification.

**Table S3.** Analyses of the characteristics of the studies

|  | | | | **Test for Overall Effect** | | | **Test for Heterogeneity** | |
| --- | --- | --- | --- | --- | --- | --- | --- | --- |
| **Items** | **Type** | **WMD or RR 95% CI** | ***Z*** | | ***P*** | ***I*²** | | ***P*** |
| age | NRS | -5.23 (-8.39, -2.07) | 3.24 | | **0.001** | 84.5% | | **<0.001** |
|  | RCT | 0.66 (-2.70, 4.01) | 0.38 | | 0.702 | <0.1% | | 0.555 |
| sex | NRS | 1.14 (1.03, 1.25) | 2.63 | | **0.009** | 69.5% | | **<0.001** |
|  | RCT | 0.97 (0.86, 1.11) | 0.41 | | 0.682 | <0.1% | | 0.398 |
| Duration of acute symptoms(h) | NRS | -1.23 (-3.58, 1.11) | 1.03 | | 0.303 | 43.9% | | **0.076** |
| Perforation size(mm) | NRS | -0.43 (-2.00, 1.15) | 0.53 | | 0.594 | 80.6% | | **0.006** |
|  | RCT | 0.59 (-0.72, 1.89) | 0.88 | | 0.378 | <0.1% | | 0.776 |
| Shock on admission | NRS | 0.75 (0.31, 1.85) | 0.62 | | 0.535 | <0.1% | | 0.924 |
|  | RCT | 0.60 (0.18, 2.02) | 0.83 | | 0.408 | <0.1% | | 0.967 |
| History of peptic ulcer | NRS | 0.95 (0.70, 1.29) | 0.31 | | 0.754 | <0.1% | | 0.647 |
| History of NAISD | NRS | 0.98 (0.39, 2.44) | 0.04 | | 0.968 | 64.9% | | **0.022** |
| History of abdominal surgery | NRS | 0.74 (0.44, 1.23) | 1.17 | | 0.234 | 7.8% | | 0.362 |

CI, confidence interval; LR, laparoscopic repair; OR, open repair; RR, relative risks; WMD, weighed mean difference; NRS, non-randomized studies; RCT, randomzed controlled trails; data in bold, significant P-value.

^a^ Number of comparisons.

**Table S4.** Results of all NRS comparing LR with OR

|  | | | | | **Test for Overall Effect** | | | **Test for Heterogeneity** | |
| --- | --- | --- | --- | --- | --- | --- | --- | --- | --- |
| **Items** | **Type** | **n**^a^ | **WMD or RR 95% CI** | ***Z*** | | ***P*** | ***I*²** | | ***P*** |
| Operative time | NRS | 19 | 18.04 (10.07, 26.00) | 4.44 | | **<0.001** | 96.9% | | **<0.001** |
| First oral intake day | NRS | 7 | -1.34(-2.00, -0.68) | 3.96 | | **<0.001** | 95.6% | | **<0.001** |
| Postoperative hospitalization | NRS | 16 | -2.66 (-3.59, -1.74) | 5.63 | | **<0.001** | 91.4% | | **<0.001** |
| Reoperation rate | NRS | 8 | 1.25 (0.66, 2.35) | 0.68 | | 0.494 | 31.5% | | 0.176 |
| Postoperative complications | NRS | 20 | 0.56 (0.38, 0.81) | 3.02 | | **0.002** | 57.7% | | **0.001** |
| mortality | NRS | 19 | 0.36 (0.26, 0.50) | 6.12 | | **<0.001** | 3.1% | | 0.419 |
| Analgesic injection (days) | NRS | 4 | -3.03 (-4.63, -1.44) | 3.72 | | **<0.001** | 97.7% | | **<0.001** |
| Analgesic injection (mg) | NRS | 4 | -103.47 (-120.02, -86.92) | 12.26 | | **<0.001** | 33.6% | | 0.211 |

CI, confidence interval; LR, laparoscopic repair; OR, open repair; RR, relative risks; WMD, weighed mean difference; NRS, non-randomized studies; data in bold, significant P-value.

^a^ Number of comparisons.

**Table S5.** Subcategory of postoperative complications comparing LR with OR

|  | | | | | **Test for Overall Effect** | | | **Test for Heterogeneity** | |
| --- | --- | --- | --- | --- | --- | --- | --- | --- | --- |
| **Items** | **Type** | **n**^a^ | **WMD or RR 95% CI** | ***Z*** | | ***P*** | ***I*²** | | ***P*** |
| Dehiscence/Fistula | NRS^b^ | 4 | 0.69 (0.15, 3.21) | 0.48 | | 0.631 | <0.1% | | 0.725 |
|  | RCT | 3 | 1.22 (0.28, 5.32) | 0.27 | | 0.789 | <0.1% | | 0.449 |
| Abscesses | NRS^b^ | 6 | 0.73 (0.30, 1.77) | 0.69 | | 0.488 | <0.1% | | 0.776 |
|  | RCT | 4 | 0.59 (0.22, 1.57) | 1.06 | | 0.289 | 48.9% | | 0.118 |
| Ileus | NRS^b^ | 6 | 0.33 (0.13, 0.83) | 2.36 | | **0.018** | <0.1% | | 0.774 |
|  | RCT | 3 | 0.41 (0.10, 1.80) | 1.18 | | 0.238 | <0.1% | | 0.981 |
| Wound infection | NRS^b^ | 8 | 0.28 (0.14, 0.56) | 3.65 | | **<0.001** | 7.0% | | 0.376 |
|  | RCT | 4 | 0.52 (0.30, 0.93) | 2.21 | | **0.027** | 26.7% | | 0.252 |
| Pneumonia | NRS^b^ | 11 | 0.74 (0.27, 2.06) | 0.58 | | 0.565 | 38.5% | | 0.093 |
|  | RCT | 4 | 0.47 (0.18, 1.24) | 1.52 | | 0.129 | 34.7% | | 0.204 |
| Urinary tract infection | NRS^b^ | 3 | 1.38 (0.30, 6.29) | 0.42 | | 0.676 | <0.1% | | 0.551 |
|  | RCT | 3 | 0.65 (0.19, 2.22) | 0.69 | | 0.488 | <0.1% | | 0.405 |
| Gastric emptying difficulty | NRS^b^ | 2 | 1.18 (0.19, 7.40) | 0.18 | | 0.859 | <0.1% | | 0.600 |
| Burst abdomen | NRS^b^ | 4 | 0.46 (0.14, 1.51) | 1.28 | | 0.200 | 19.9% | | 0.290 |
| Incisional hernia | RCT | 4 | 0.41 (1.56, 1.08) | 1.81 | | 0.070 | <0.1% | | 0.989 |

CI, confidence interval; LR, laparoscopic repair; OR, open repair; RR, relative risks; NRS, non-randomized studies; RCT, randomzed controlled trails; data in bold, significant P-value.

^a^ Number of comparisons.

^b^ High quality NRS (≥7 scores).

1

**Figure legend**

Fig S1. Galbraith plot for investigating the source of heterogeneity. The studies outside the parallel lines were considered contributing to the heterogeneity. (A) Operative time, (B) First oral day, (C) Postoperative hospitalization, (D) Postoperative complications.

Fig S1


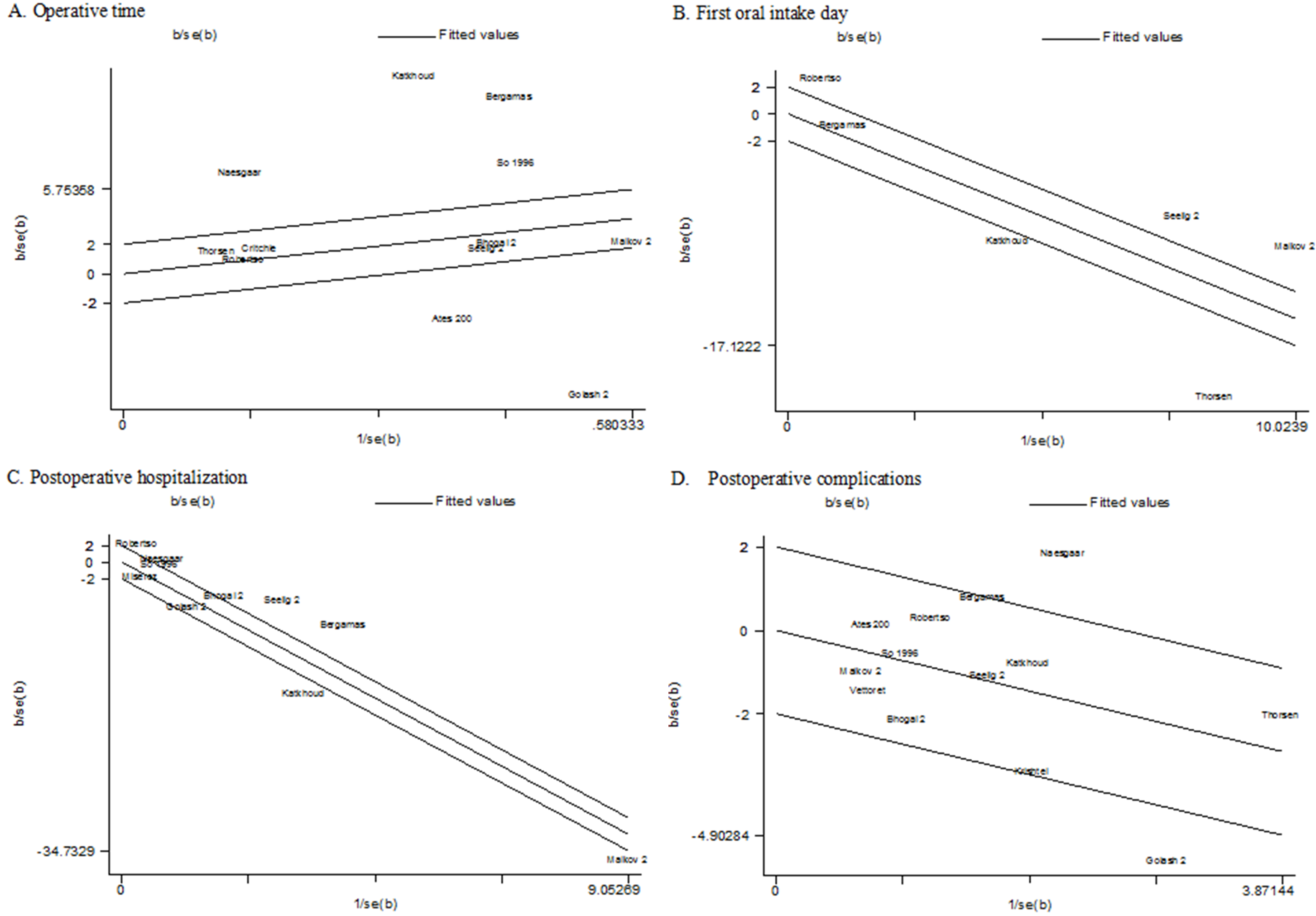

Supplement: Supplementary Information [file srep13976-s1.doc]
